# Evaluation of Antibacterial and Cytotoxic Potency of Polyherbal Gel Formulation Containing *Achyranthes Aspera* and *Trachyspermum Ammi* as Intracanal Medicament: An *in vitro* Study 

**DOI:** 10.30476/dentjods.2024.100380.2218

**Published:** 2025-06-01

**Authors:** Ram Surath Kumar, Anil V Ankola, Mahantesh B. Nagamoti, Roopali M. Sankeshwari, Kishori P. Sutar, Shushant I Jigan, Atrey J Pai Khot, Ritiha Uppin

**Affiliations:** 1 Dept. of Public Health Dentistry, KLE Vishwanath Katti Institute of Dental Sciences, KLE Academy of Higher Education and Research, Belagavi- 590010, India.; 2 Dept. of Microbiology, Jawaharlal Nehru Medical College, KLE Academy of Higher Education and Research, Belagavi- 590010, India.; 3 Dept. of Pharmaceutics, KLE College of Pharmacy, KLE Academy of Higher Education and Research, Belagavi- 590010, India.; 4 Dr. Prabhakar Kore Basic Science Research Center, KLE Academy of Higher Education and Research, Belagavi- 590010, India.

**Keywords:** Anti-infective agents, Calcium hydroxide, Chlorhexidine, Endodontics, Herbal

## Abstract

**Background:**

The overwhelming increase of antibiotic-resistant bacteria, and the adverse reactions of using synthetic drugs such as chlorhexidine (CHX) and calcium hydroxide (Ca(OH_2_))- based intracanal medicaments have made it mandatory to search for effective substitutes. Herbal medicines like *Achyranthes Aspera* (*A.aspera*) and *Trachyspermum Ammi* (*T.ammi*) have been used in many clinical conditions and it appears to be a distinct material next to Ca(OH_2_) in the field of dentistry.

**Purpose:**

Evaluate the antibacterial potential and cytotoxic effects of novel polyherbal gel containing *A.aspera* and *T.ammi*, CHX gel, and Ca(OH_2_) paste based intracanal medicaments in root canal treatment against *Enterococcus faecalis* (*E.faecalis*).

**Materials and Method:**

In this *in vitro* study, Ethanolic extracts of *A.aspera* and *T.ammi* were prepared by the Soxhlet apparatus method. The individual plant extracts and the plant extract mixtures (1:1, 2:1, and 1:2), CHX, and Ca(OH_2_) were assessed for minimum inhibitory concentration (resazurin microtiter assay), fractional inhibitory concentration and minimum bactericidal concentration (spread plating method) against *E.faecalis*. The polyherbal intracanal medicament was assessed for zone of inhibition (well diffusion method) and cytotoxicity ( MTT assay) on human periodontal ligament cells. All experiments were performed in triplicate.

**Results:**

Polyherbal gel containing *A.aspera* and *T.ammi*, CHX gel, and Ca(OH_2_) paste-based intracanal medicaments showed statistically significant antibacterial activity (*p* <0.05) against *E.faecalis* with CHX showing superior properties followed by polyherbal gel. The results of the cytotoxicity assay demonstrated the good biocompatibility of the polyherbal intracanal medication, which exhibited 95.13% of surviving cells.

**Conclusion:**

The use of herbal alternatives as an intracanal medicament proved to be advantageous considering the several undesirable characteristics of CHX and Ca(OH_2_).

## Introduction

The success of root canal treatment mainly depends on the complete elimination of microorganisms from the root canal system [ 1 ]. The best way to accomplish this goal is to combine biomechanical preparation with different intracanal medicaments and root canal irrigants [ 2 ]. *Enterococcus faecalis* (*E.faecalis*), a gram-positive facultative anaerobic can survive as a single microorganism or as a substantial fraction of the root canal flora. It is the major cause of endodontic failure and resistant infections resulting in periradicular lesions. *E.faecalis* is common in periradicular lesions following failed root canal treatment (29-77%) because of its antibiotic resistance [ 3 ].

Chlorhexidine (CHX) has been suggested as an intracanal medicament because of its antimicrobial property and substantivity. It is successful at eliminating *E.faecalis* from the root canals and dentinal tubules [ 4 ]. Furthermore, CHX is harmful to vital tissues, and its toxic effects increase with concentration. Calcium hydroxide (Ca(OH_2_)) is commonly employed as an intracanal medicament because of its bactericidal effects. Its high pH of 12.5 is detrimental to protein structures and cell membranes of endodontic pathogens [ 5 ]. It has limited penetration into dentinal tubules and is ineffective against all endodontic pathogens, including *E.faecalis* and its endotoxins. Allergic responses, toxicity, and resistance are a few reported negative impacts linked to the usage of Ca(OH)_2_ [ 6 ]. Nonetheless, none of them is capable of completely eliminating resistant microorganisms [ 7 ].

The overwhelming increase of antibiotic-resistant bacteria and the adverse reactions of using synthetic drugs such as CHX and Ca(OH_2_) as intracanal medicaments have made it mandatory to search for effective substitutes. Traditional medicine has gained popularity due to its affordability, therapeutic value, and reputation for having fewer side effects as compared to synthetic drugs [ 8 ]. *Achyranthes Aspera Linn* (Apamarga) belongs to the family Amaranthaceae. The active ingredients of *Achyranthes Aspera* (*A.aspera* )are utilized as an antibacterial, antifungal, antiviral, antimalarial, antiarthritic, antileprotic, antispasmodic, purgative, diuretic, oestrogenic, and cardiotonic agent [ 9 ]. Fresh *A.aspera* root was used as a toothbrush in routine oral hygiene practices in ancient times [ 9 ]. *Trachyspermum Ammi Linn* (Ajwain), belongs to the family Apiaceae. The seeds have therapeutic benefits in medicine including aphrodisiac properties, analgesic, antibacterial, antiviral, antifungal, antioxidant, and anti-inflammatory activity [ 10 ].

There is an impending need to bring to light the medicinal properties of such herbal products. Using herbal intracanal medicament as a suitable alternative can be a breakthrough for these problems, thereby increasing the success rate of root canal treatment. The present study aims to evaluate the antibacterial potential and cytotoxic effects of novel polyherbal gel containing *A.aspera* and *Trachyspermum Ammi* (*T.ammi*), CHX gel and Ca(OH_2_) paste based intracanal medicaments in root canal treatment against *E.faecalis*. 

## Materials and Method

### Collection and authentication of plant specimen

The current study was an experimental *in vitro* study and was in accordance with Good Laboratory Practice standards [ 11 ]. Roots of *A.aspera* and seeds of *T.ammi* were obtained from the Ayurveda pharmacy of a recognized institute, Belagavi, India. A taxonomist from the Indian Council of Medical Research - National Institute of Traditional Medicine, conducted authentication of the specimen (deposition number: RMRC-1618).

### Preparation of plant extracts

**Figure 1:**
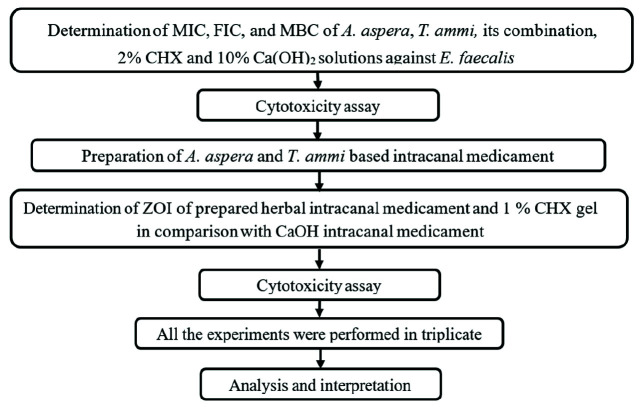
Study flow diagram, Minimum inhibitory concentration (MIC); Fractional inhibitory concentration (FIC); Minimum bactericidal concentration (MBC); Plant extract mixture (PEM) of *Achyranthes aspera* (*A.aspera*) and *Trachyspermum ammi* (*T.ammi*) in the ratio of 1:1 (w/v), 2:1 (w/v), 1:2 (w/v); 10% Calcium hydroxide (Ca(OH)_2_) solution; 2% Chlorhexidine (CHX) solution; Zone of inhibition (ZOI)

Fresh roots of *A.aspera* and seeds of *T.ammi* were dried under shade and ground into a coarse powder. Ethanol extracts of *A.aspera* and *T.ammi* were prepared by the Soxhlet apparatus method in 600 mL of solvent (Changshu Hongsheng Fine Chemicals Ltd, China) at 50ºC. *A.aspera* and *T.ammi* extracts were prepared after the cyclic procedure with duration of 8 hours and 5.5 hours, respectively. The cycles were repeated until the solvent transformed from a coloured to a colourless one. A total of 150 grams of coarse powders were utilized in 600 mL of solvent each in a 1:4 ratio to produce an extraction yield of 13.9 grams of *A.aspera* (9.3%) and 30.2 grams (20.1%) of *T.ammi* crude extracts. The extracts were removed from the apparatus and dried in the IKA RV 10 rotary flash evaporator [ 12 - 13 ]. The prepared crude extracts were subjected to preliminary phytochemical screening using the standard procedures as suggested by Evans *et al*. [ 14 ] (Table 1). The sterile extracts were stored at -20°C for further use. Figure 1 shows the methodology adopted for conducting the study.

**Table 1:** Phytochemical Screening of the ethanolic extracts of *Achyranthes aspera* and *Trachyspermum ammi*

Extract name	Test done	Observation	Inference
*Achyranthes aspera*	Flavonoids		
	Sulphuric acid test	A deep yellow solution was observed	+
	Lead acetate tests	A yellow precipitate was observed	+
	Alkaloids		
	Dragendoff’s test	An orange-brown precipitate was observed	+
	Meyer’s test	Precipitate was observed	+
	Tannins and Phenolic compounds		
	Ferric chloride test	A deep blue-black colour was observed	+
	Lead acetate	A white precipitate was observed	+
	Steroid		
	Salkowski’s reaction	An appearance of red chloroform layer or greenish-yellow fluorescence in the acid layer	+
	Liebermann’s test	No appearance of blue colour	-
*Trachyspermum ammi*	Flavonoids		
	Lead acetate tests	A yellow precipitate was observed	+
	Alkaloids		
	Meyer’s test	Precipitate was observed	+
	Tannins and Phenolic compounds		
	5 % Ferric chloride	A deep blue-black colour was observed	+
	Steroid		
	Salkowski’s reaction	An appearance of red chloroform layer or greenish-yellow fluorescence in the acid layer	+
	Volatile oil		
	Solubility test in 90 % Alcohol	Soluble in Chloroform	+

Positive (+): Present; Negative (-): Absent

### Source and preparation of bacterial suspension

For a fresh standard strain of *E.faecalis*, ATCC 29212 was procured from HiMedia Laboratories Pvt. Ltd., India. Direct colony suspension of the bacterial isolate was prepared anaerobically in 5mL Brain-Heart Infusion (BHI) broth (HiMedia Pvt. Ltd., India) and inoculated in an anaerobic chamber (N_2_ 80%, H_2_ 10%, CO_2_ 10%) for 48 hours at 37°C. The turbidity was adjusted to 0.5 McFarland Standard (1x10^8^ Colony Forming Unit (CFU)/mL).

### Preparation of stock solutions, and CHX and Ca(OH_2_) solutions

Each of the sterile extracts (w/v) weighing 50mg was reconstituted in 1000µL of 10% Dimethyl sulfoxide (DMSO) (Qualigens, Thermo Fisher Scientific Pvt. Ltd, India). A MixMate^®^ Vortex agitator (Eppendorf, Australia) was used to agitate the mixture for 3 minutes at 1000 rpm and then bath sonicated using Branson bath sonicator 1800 (Branson Ultrasonics, Danbury, CT) for 15 minutes. A solution of 2% CHX (w/v) was prepared using CHX hydrochloride salt BP grade (ICPA Health Products Ltd., India). A solution of 10% Ca(OH_2_) (w/v) which used as a positive control was prepared using Ca(OH_2_) powder (Molychem Pvt Ltd., India) [ 15 ].

### Minimum inhibitory concentration (MIC) and Fractional inhibitory concentration (FIC)

A Resazurin microtiter assay, using the standard protocol of the Clinical and Laboratory Standards Institute and a modified approach of Pai Khot *et al*. [ 16 ], were employed in determining the minimum inhibitory concentration (MIC) of individual component extracts, plant extract mixtures (PEMs) [in the ratios of 1:1, 2:1, and 1:2 (w/v)], 2% CHX solution, and 10% Ca(OH_2_) against *E.faecalis*. 

A volume of 100 µL of the test sample (*A.aspera*) was introduced to the first well of the 96-well microtiter plate (NEST-Biotechnology, China) followed by 100 µL of sterile BHI broth was introduced to each well. The various concentrations (50-0.19 mg/mL) of extract were prepared by a serial doubling dilution method. This resulted in a 50% reduction in subsequent well concentrations. Finally, 10 µL of standardized bacterial suspension was added to the respective wells. In addition, one well filled with 200 µL of BHI broth served as a vehicle control to confirm there was no contamination during plate preparation. In a separate well containing 200µL BHI broth, 10µL of bacterial suspension was added that acted as a growth control. The plates were sealed and incubated in McIntosh and Fildes’ anaerobic jar using the microaerophilic atmosphere generation system at 37°C for 48 hours. After the period of incubation, 10 µL of resazurin solution (Hi-Cert™ HiMedia^®^ Laboratories, Pvt. Ltd, India) (0.5mg/mL) was added to each well and further incubated for 4 hours in anaerobic condition at 37°C. The resulting change in resazurin blue/purple colour to pink/red was inferred as an indication of bacterial growth, and no colour change indicates inhibition of bacterial growth. The MIC was taken as the lowest concentration with no change in resazurin colour [ 17 ]. The experiment was repeated for other plant extracts (*T.ammi* and PEMs). The experiments were performed in triplicate. To assess the synergy of the extracts, the fractional inhibitory concentration (FIC) was determined using the following formula:

FICa(FIC of A.aspera)=MIC of PEM MIC of A.aspera alone 

FICb(FIC of T.ammi)=MIC of PEM MIC of T.ammi alone 

The FIC index (ΣFIC) formula: $\mathrm{\Sigma FIC}=\frac{1}{2}({\mathrm{FIC}}_{a}+{\mathrm{FIC}}_{b})$

FIC index establishes the interaction among various extracts in the PEM wherein is evaluated using the following value range: value < 0.5 as synergistic, > 0.5-1 as additive, >1-4 as no interaction and value > 4 as antagonistic [ 18 ].

### Minimum Bactericidal Concentration (MBC)

MBC was determined by the spread plating method. Bacterial suspension of 20µL from the wells with a concentration higher than the MIC value was subjected to inoculation on plates containing BHI agar (HiMedia Pvt. Ltd., India) and incubated for 24 hours at 37℃. The lowest concentration of the extract (*A.aspera*) that showed no growth was taken as MBC [ 19 ]. The experiments were repeated for *T.ammi*, PEM (1:1), and 2% CHX solution in comparison to 10% Ca(OH_2_) based on the findings of MIC. The experiments were performed in triplicate.

### Formulation of herbal intracanal medicament

**Figure 2:**
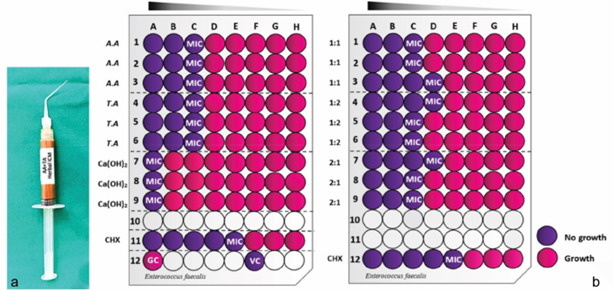
**a:** Prepared *Achyranthes aspera* (A.A) and *Trachyspermum ammi* (T.A) gel-based herbal intracanal medicament, **b:** Schematic diagram of resazurin microtiter assay for determining Minimum Inhibitory Concentration (MIC) of individual component plant extracts, and plant extract mixtures (PEMs in 1:1, 1:2, and 2:1), 2% chlorhexidine (CHX) and 10% calcium hydroxide (Ca(OH)_2_) solutions against *Enterococcus faecalis*. Growth control (GC); Sterility control (VC). Serial dilution of the plant extracts starts at a concentration of 50 mg/mL; CHX starts at a concentration of 1.2 mg/mL, Ca(OH)_2_ starts at a concentration of 100 mg/mL.

Polyherbal intracanal medicament was prepared with a weighed proportion of extracts comprising 5% *A.aspera* and 5% *T.ammi* (w/v) mixed with 2% glycerine. Methylparaben (0.5%), ethylparaben (0.01%), and sodium benzoate (0.5%) were dissolved in 4mL of deionized distilled water. Finally, 2.5% of sodium carboxymethylcellulose was added which was kept for hydration for 24 hours to obtain the desired 5mL of intracanal medicament gel formulation (Figure 2a).

### Antibacterial susceptibility testing

Prepared polyherbal gel, 1% CHX gel (Hexigel, ICPA Health Products Ltd., India) and Ca(OH_2_) paste (ApexCal^®^, Ivoclar Vivadent, Liechtenstein) based intracanal medicaments were tested using an agar well diffusion assay. The antibacterial susceptibility testing was carried out as per Valgas *et al*. [ 20 ]. On a BHI agar plate, colonies of microorganisms were inoculated with sterile cotton swabs, swabbed three times, and adjusted to McFarland 0.5 turbidity. A sterile borer was used to create a 6mm diameter and 4mm depth well in the inoculated plates. Four wells on each BHI agar plate were prepared, one each for polyherbal gel, CHX gel, Ca(OH_2_) paste, and negative control (Nucleus-free water, Qiagen, Germany). Each well received 100μL of the respective test compounds. The zone of inhibition (ZOI) was determined with a vernier calliper (Scienceware, Pequannock, NJ) after 48 hours of incubation at 37°C. The experiments were performed in triplicate.

### Cytotoxicity assay

The MTT (3-[4,5-dimethylthiazol-2-yl]-2,5 diphenyl tetrazolium bromide) (HiMedia^®^ Laboratories, Pvt. Ltd, India) cytotoxicity assay was performed on adult human periodontal ligament (PDL) cells with the Institutional Ethics Committee's approval (reference number: 1511, dated: 28.11.2021). The patient gave his/her written consent after being informed. The cells were harvested from the healthy periodontal tissue of a premolar extracted for orthodontic purposes. The cytotoxicity assay was carried out as per Van Meerloo *et al*. [ 21 ]. *in vitro* growth inhibition effect of the test sample was calculated using ELISA (Epoch, BioTek^®^ Instruments, Inc., USA) by conversion determination of MTT into Formazan blue by living cells. Each well was seeded with 50µL of 4000 cells/mL cell suspension with the addition of Dulbecco's Modified Eagle Medium (Gibco™ Life Technologies, India) to get the final volume of 150 µL. In the presence of 5 % CO2, the respective test sample (100 µL each) was introduced to the wells and incubated in a CO2 incubator (New Brunswick™ Galaxy^®^ 170 R, Eppendorf, Germany) for 24hours at 37°C. About 20µL of 5mg/mL MTT reagent was introduced to the wells after 24hours. The supernatant was removed without disrupting the precipitated Formazan crystals. DMSO (100µL) was added to the crystals to dissolve them. The optical density (OD) was measured at a wavelength of 570nm and the experiment was performed in triplicate [ 21 ].

### Statistical analysis

The collected data were input into Microsoft Excel (2020) and analyzed with SPSS^®^, IBM Corp. Released 2012 IBM SPSS, and Version 21.0. Armonk, NY. The descriptive data were given in the form of mean±standard deviation. To compare the difference in the antibacterial and cytotoxic properties of extracts, polyherbal gel, CHX gel, and Ca(OH_2_) paste based intracanal medicaments, Kruskal-Wallis test was used followed by Dunn's *post hoc* test. Statistical significance was set at *p*≤ 0.05. 

## Results

The mean MIC, FIC, and MBC of individual extract and PEMs (1:1, 2:1 and 1:2) against *E.faecalis* are summarized in Table 2. It was also noted that the growth of *E.faecalis* was inhibited at the higher concentration of 12.5mg/mL for the individual plant extracts and the PEMs. *E.faecalis* was most sensitive to 2% CHX solution at 0.15mg/mL. In contrast, the bacteria were the least sensitive to 10% Ca(OH_2_) solution, requiring a higher concentration of 100 mg/mL (Figure 2b). According to ΣFIC, the interaction of the plant extracts in the PEMs was found to be additive for *E.faecalis* with a ΣFIC of 0.83.

**Table 2:** Determination of MIC, FIC, and MBC of *Achyranthes aspera* and *Trachyspermum ammi* extracts, plant extract mixtures, 2% Chlorhexidine and 10% Calcium hydroxide solutions against Enterococcus faecalis

Minimum inhibitory concentration (mg/mL)								Fractional inhibitory concentration index									
*A. aspera* extract a	*T. ammi* extract b	PEM (w/v)			CHX solution	Ca(OH_2_) solution	Statistics	PEM									Interaction
		1:1	1:2	2:1			*p* Value	1:1		2:1		1:2		1:1	2:1	1:2	
								FIC_a_	FIC_b_	FIC_a_	FIC_b_	FIC_a_	FIC_b_	ΣFIC			
12.50^α^	12.50^α^	10.43^α^	10.43^α^	10.43^α^	0.15^β^	100^γ^	0.012^*^	0.83	0.83	0.83	0.83	0.83	0.83	0.83	0.83	0.83	Additive interaction
Minimum bactericidal concentration (mg/mL)																	
*A. aspera* >extract	*T. ammi* extract	1:1 PEM (w/v)			CHX solution	Ca(OH_2_) solution	*p* Value										
25.00^α^	25.00^α^	25.00^α^			0.08^β^	100^γ^	0.007^*^										

Minimum inhibitory concentration (MIC); Fractional inhibitory concentration (FIC); MBC: Minimum bactericidal concentration (MBC); Plant extract mixture (PEM) of *Achyranthes aspera* (*A.aspera*) and *Trachyspermum ammi* (*T.ammi*) in the ratio of 1:1 (w/v), 2:1 (w/v), 1:2 (w/v); 10% Calcium hydroxide (Ca(OH)_2_) solution; 2% Chlorhexidine (CHX) solution. FICa: FIC of *A.aspera*; FICb: FIC of *T.ammi*. ΣFIC = 1/2(FIC_a_ + FIC_b_); The FIC index was interpreted as synergistic interactions (if < 0.5), ¥ additive (if in the range of > 0.5-1) and no interaction (if in the range > 1-4), or as an antagonist (if > 4). The results are shown as average values of triplicate. Different lowercase (α, β, γ) indicate a significant difference between extracts, CHX and Ca(OH_2_) solutions within the same row. The statistical test used: Dunn’s *post hoc* method following a significant Kruskal-Wallis test; level of significance: *p ≤ 0.05 is considered statistically significant.

**Figure 3:**
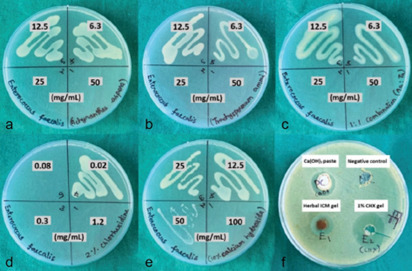
Photographs showing Minimum Bactericidal Concentration (MBC) of **a:** *Achyranthes Aspera* extract and **b:** *Trachyspermum Ammi* extracts, **c:** Plant extract mixture (PEM) in the ratio of 1:1, **d:** 2% Chlorhexidine solution and **e:** 10% Calcium hydroxide solution, **f:** Culture specimen for agar well diffusion method against *Enterococcus faecalis*, 1% chlorhexidine (CHX) gel; 10% calcium hydroxide (Ca(OH)_2_) paste. The serial numbers on each BHI agar plate corresponded to the columns of the 96-well plate for each panel.

The MBC findings revealed that individual plant extracts and PEM (1:1) inhibited colony formation of *E.faecalis* at a higher concentration of 25mg/mL, whereas CHX solution inhibited at a higher concentration of 0.08mg/mL. Ca(OH_2_) solution represented a weak antibacterial effect at 100mg/mL (Figure 3a-e). There was a statistically significant difference between the groups (*p*= 0.007).

**Figure 4:**
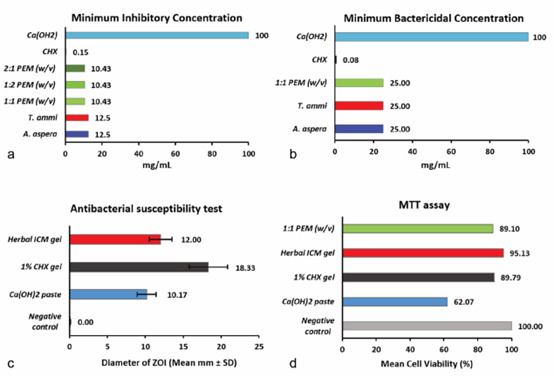
Graphical representation results of **a:** Minimum Inhibitory Concentration (MIC); **b:** Minimum Bactericidal Concentration (MBC); **c:** Antibacterial susceptibility test and **d:** MTT cytotoxicity assay. Plant extract mixture (PEM) of *Achyranthes aspera* and *Trachyspermum ammi* in the ratio of 1:1 (w/v), 2:1 (w/v), 1:2 (w/v); Calcium hydroxide (Ca(OH)_2_); Chlorhexidine (CHX)

The maximum ZOI for polyherbal gel (12.0±1.5mm) for *E.faecalis* was smaller than 1% CHX gel (18.3±2.5 mm) but larger than Ca(OH_2_) paste (10.2±1.3mm). The antibacterial susceptibility difference between the intracanal medicament groups was statistically significant; *p*= 0.018 (Table 3 and Figure 4).

**Table 3:** Determination of zone of inhibition against *Enterococcus faecalis* and the comparison of optical densities of surviving cells of various test compounds at a wavelength of 570 nm

Test performed	Intracanal medicaments			Negative control	1:1 PEM (w/v)	Statistics *p* Value	Results as observed
	Herbal intracanal medicament gel	1% CHX gel	Ca(OH)_2_ paste				
Antibacterial susceptibility test							
Diameter of ZOI (Mean mm ± SD)	12.00±1.50	18.33±2.52	10.17±1.26	0.00±0.0	-	0.018^*^	Significant antibacterial susceptibility difference between groups
MTT cytotoxicity assay							
Optical Density (Mean± SD)	0.28±0.08	0.26±0.06	0.18±0.02	0.29±0.14	0.26±0.03	0.367	No cell lysis
Mean Cell Viability (%)	95.13%	89.79%	62.07%	100.00%	89.10%		

Zone of inhibition (ZOI); Intracanal medicament gel containing *Achyranthes aspera* and *Trachyspermum ammi* in ratio 1:1 (w/v); Chlorhexidine (CHX) gel; Calcium hydroxide (Ca(OH)2) paste (positive control); Nucleus-free water (negative control); Plant extract mixture (PEM). The results are shown as average values of triplicate. The statistical test used: Kruskal-Wallis test; level of significance: *p ≤ 0.05 is considered statistically significant.

The cytotoxicity assay that was carried out on adult human PDL cells revealed that polyherbal intracanal medicament exhibited 95.1% of surviving cells which demonstrated good biocompatibility when compared with Ca(OH_2_) paste (62.1% cell viability) and CHX gel (89.8% cell viability). The results were statistically insignificant (*p*= 0.367) (Table 3). 

## Discussion

*E. faecalis* represents the most prevalent Enterococcus species surviving in treated root canals and it is resistant to standard antibiotics [ 22 ]. *E. faecalis* can indeed survive in environments where nutrition availability is limited. Despite thorough biomechanical preparation and intracanal medication, this bacterium can colonize into the dentinal tubules and reinfect the obturated root canals [ 23 ]. Overall, the results confirmed that *A.aspera* and *T.ammi*-based intracanal medicament proved successful at inhibiting *E.faecalis*. Besides this, Ca(OH)_2_ represented a weak antibacterial activity. This finding is consistent with previous studies reported that Ca(OH)_2_ has broad antibacterial properties against prevalent endodontic pathogens but not against *E.faecalis*. It was also demonstrated that Ca(OH)_2_ failed to remove *E.faecalis* from dentinal tubules . *E.faecalis* remained viable in the dentinal tubules following an extended duration of Ca(OH)_2_ medication, according to Safavi *et al*. [ 25 ]. The low diffusion rate, low solubility, high density of biofilm formation, variation in alkaline potential of different formulations, and the escape of *E.faecalis* from hydroxyl ions due to attempting to hide in irregularities and the canal isthmus can all be linked to the reduced influence of Ca(OH)_2_ [ 26 ]. Furthermore, *E.faecalis* has a proton pump, which can acidify the cytoplasm. It allows the microorganism to be resistant to killing by Ca(OH)_2_ at pH 11.1 or lower and to exist for 12 months under starvation [ 27 ].

In the present study, *A.aspera* and *T.ammi*-based intracanal medicament was more effective as an antibacterial agent compared to Ca(OH)_2_ paste but less effective than CHX gel. The findings were similar to those of Basrani *et al*. [ 28 ] and Vaghela *et al*. [ 29 ] who found that CHX gel exhibited better antibacterial activity than Ca(OH)2. To overcome such drawbacks, extensive research is being conducted in the field of alternative medicine. There has been a huge turnaround towards herbal remedies in recent times.

The reason to choose *A.aspera* and *T.ammi* was their long-standing history as effective antimicrobial and anti-inflammatory agents. Several studies reported that *A.aspera* [ 30 - 31 ] and *T.ammi* [ 32 - 33 ] exhibited antibacterial and antifungal activity against various oral microorganisms like Streptococcus mutans, Lactobacillus acidophilus, *E.faecalis* and Candida albicans. In addition to this, Gokhale *et al*. [ 34 ] reported the anti-inflammatory effects of *A.aspera* in inbred Wistar rats and Swiss albino mice. In this study, and in line with two previous studies [ 35 - 36 ], *T.ammi* demonstrated significant antibacterial activity against *E.faecalis*. Recent published *in vitro* [ 37 ] and clinical trial-based [ 38 ] studies reported that the combined use of *A.aspera* and *T.ammi* exhibits antibacterial activity against specific periodontal pathogens.

In this study, FIC, MBC, and ZOI were used to assess the antibacterial properties of PEM and polyherbal intracanal medicament in place of culture techniques. The FIC index is viewed as the gold standard for determining the interaction between various natural products [ 18 ]. Focusing on single target compounds including herbal products does not generate long-term alternatives to antimicrobial resistance. Resistance to crude extracts is less common than resistance to single actives. As a result, research focusing on antimicrobial combinations can lead to a breakthrough that addresses the growing potential problem concerning antimicrobial resistance [ 18 ]. It was found that PEMs have a lower MIC than their individual constituent plant extracts. This indicates that PEM is more effective bacterial growth inhibitor, which could be due to the combined effect of bioactive substances present in the individual extracts, an additive interaction was observed as reflected by the FIC index. However, a similar MBC value as PEM indicates that it exhibits a similar bactericidal effect against *E.faecalis*.

Previous studies observed that preliminary phytochemical analysis of both extracts indicated the presence of flavonoids, alkaloids, tannins, phenolic compounds and steroids [ 39 - 40 ]. Flavonoids present in *A.aspera* are responsible for antibacterial activity, according to Pandey *et al*. [ 41 ]. Moreover, Modareskia *et al*. [ 42 ] revealed that the major constituents in *T.ammi* were phenolic compounds (Thymol- 59.9-96.4%, p-cymene- 0.6-21.2%, γ-terpinene- 0.2-17.8%, and carvacrol- 0.4-2.8%) exhibit strong antibacterial activity.

The cytotoxicity assay findings confirmed that *A.aspera* and *T.ammi*-based intracanal medicament exhibited the highest surviving cells, which proved good biocompatibility. Furthermore, low concentrations of extracts were used in the formulation of polyherbal intracanal medicament, thus considering the safety concerns of using these extracts in humans. Herbal alternatives are frequently employed because of their wide range of advantages, including ease of availability, simplicity of cultivation and processing, acceptance, low toxicity, cost-effectiveness, and lack of microbial resistance.

Although the findings support that this novel polyherbal intracanal medicament may serve as an effective and biocompatible antibacterial agent, it is worth noting that possible interactions between the physical, chemical, and pharmacological properties of *A.aspera* and *T.ammi* with dentinal tubules remain unknown. In light of the present study, a robust experimental model remains to be investigated in order to evaluate the long-term antibacterial efficacy of *A.aspera* and *T.ammi*-based intracanal medicament. Further investigations in animal or human models are needed to conclusively recommend herbal gel as an intracanal medicament. 

## Conclusion

*A.aspera*- and *T.ammi*- based polyherbal intracanal medicaments and CHX gel demonstrated superior antibacterial activity against *E.faecalis*. The polyherbal intracanal medicament is a promising therapeutic agent with good biocompatibility over CHX and Ca(OH)2. The use of herbal alternatives as an intracanal medicament proved to be advantageous considering the several undesirable characteristics of CHX and Ca(OH)2.Acknowledgment

The authors would like to thank Suneel Dodamani, Shivani Tendulkar, Vinuta Hampiholi, Viswanath and Preetam Mehetri for providing subject insights during the course of the study. We would also like to acknowledge Dr. Prabhakar Kore, Basic Science Research Centre for providing the facilities for successfully carrying out our experiment in the tissue culture laboratory. We also thank Jayapriya T for the English language editing.

## References

[1] Gomes BP, Drucker DB, Lilley JD ( 1994). Association of specific bacteria with some endodontic signs and symptoms. Int Endod J.

[2] Akcay M, Arslan H, Topcuoglu HS, Tuncay O ( 2014). Effect of Calcium Hydroxide and Double and Triple Antibiotic Pastes on the Bond Strength of Epoxy Resin-based Sealer to Root Canal Dentin. J Endod.

[3] Siqueira JF, Rôças IN ( 2004). Polymerase chain reaction-based analysis of microorganisms associated with failed endodontic treatment. Oral Surg Oral Med Oral Pathol Oral Radiol Endod.

[4] Gomes BPF de A, Vianna ME, Sena NT, Zaia AA, Ferraz CCR, Filho FJ de S ( 2006). In vitro evaluation of the antimicrobial activity of calcium hydroxide combined with chlorhexidine gel used as intracanal medicament. Oral Surg Oral Med Oral Pathol Oral Radiol Endod.

[5] Vasudeva A, Sinha DJ, Tyagi SP, Singh NN, Garg P, Upadhyay D ( 2017). Disinfection of dentinal tubules with 2% Chlorhexidine gel, Calcium hydroxide and herbal intracanal medicaments against Enterococcus faecalis: An in vitro study. Singapore Dent J.

[6] Mohammadi Z, Shalavi S, Yazdizadeh M ( 2012). Antimicrobial Activity of Calcium Hydroxide in Endodontics: A Review. Chonnam Med J.

[7] Maekawa LE, Valera MC, Oliveira LD de, Carvalho CAT, Koga-Ito CY, Jorge AOC ( 2011). In vitro evaluation of the action of irrigating solutions associated with intracanal medications on Escherichia coli and its endotoxin in root canals. J Appl Oral Sci Rev FOB.

[8] Verma S, Singh SP ( 2008). Current and future status of herbal medicines. Veterinary World.

[9] Vetrichelvan T, Jegadeesan M ( 2003). Effect of alcohol extract of Achyranthes aspera Linn on acute and subacute inflammation. Phytother Res.

[10] Bairwa R, Sodha RS, Rajawat BS ( 2012). Trachyspermum ammi. Pharmacogn Rev.

[11] Kendall G, Bai R, Błazewicz J, De Causmaecker  P, Gendreau M, John R, et al ( 2016). Good laboratory practice for optimization research. J Oper Res Soc.

[12] Teshome D, Tiruneh C, Berhanu L, Berihun G, Belete ZW ( 2021). Developmental Toxicity of Etha nolic Extracts of Leaves of Achyranthes aspera, Amaranthaceae in Rat Embryos and Fetuses. J Exp Pharmacol.

[13] Pai Khot AJ, Ankola AV, Naik VV, Sankeshwari RM, Kumar RS, Shah MA ( 2023). Remineralising potential of Ocimum basilicum varnish and fluoride varnish on initial enamel caries: An in vitro microscopic study. J Oral Maxillofac Pathol.

[14] Evans WC, Evans D, Trease GE (2002). Trease and Evans Pharmacognosy.

[15] Ferreira CM, Rosa OP da S, Torres SA, Ferreira FB de A, Bernardinelli N ( 2002). Activity of endodontic antibacterial agents against selected anaerobic bacteria. Braz Dent J.

[16] Pai Khot AJ, Ankola AV, Dodamani S, Sankeshwari RM, Kumar RS, Santhosh VN ( 2023). Assessment of potential antimicrobial activity of Ocimum basilicum extract and chlorhexidine against Socransky’s complex pathogens of oral cavity: An in vitro study. J Indian Soc Periodontol.

[17] Sarker SD, Nahar L, Kumarasamy Y ( 2007). Microtitre plate-based antibacterial assay incorporating resazurin as an indicator of cell growth, and its application in the in vitro antibacterial screening of phytochemicals. Methods.

[18] Vuuren S Van, Viljoen A ( 2011). Plant-Based antimicrobial studies- methods and approaches to study the interaction between natural products. Planta Med.

[19] MacFarlane TW, Samaranayake LP (2014). Clinical oral microbiology.

[20] Valgas C, Souza SM de, Smânia EFA, Smânia Jr A ( 2007). Screening methods to determine antibacterial activity of natural products. Braz J Microbiol.

[21] Van Meerloo  J, Kaspers GJL, Cloos J ( 2011). Cell sensitivity assays: The MTT assay. Methods Mol Biol.

[22] Pinheiro ET, Gomes BPFA, Ferraz CCR, Teixeira FB, Zaia AA, Souza Filho FJ ( 2003). Evaluation of root canal microorganisms isolated from teeth with endodontic failure and their antimicrobial susceptibility. Oral Microbiol Immunol.

[23] Love RM ( 2001). Enterococcus faecalis- a mechanism for its role in endodontic failure. Int Endod J.

[24] Kumar RS, Ankola AV, Sankeshwari RM, Hebbal M, Hampiholi V, S LK, et al ( 2023). Effectiveness of various irrigant activation techniques on the penetration of sodium hypochlorite into lateral canals of mature permanent teeth: A systematic review and meta-analysis. Saudi Dent J.

[25] Safavi KE, Spngberg LSW, Langeland K ( 1990). Root canal dentinal tubule disinfection. J Endod.

[26] Gomes BPFA, Drucker DB, Lilley JD ( 1994). Association of specific bacteria with some endodontic signs and symptoms. Int Endod J.

[27] Evans M, Davies JK, Sundqvist G, Figdor D ( 2002). Mechanisms involved in the resistance of Enterococcus faecalis to calcium hydroxide. Int Endod J.

[28] Basrani B, Tjäderhane L, Santos JM, Pascon E, Grad H, Lawrence HP, et al ( 2003). Efficacy of chlorhexidine- and calcium hydroxide-containing medicaments against Enterococcus faecalis in vitro. Oral Surg Oral Med Oral Pathol Oral Radiol Endod.

[29] Vaghela DJ, Kandaswamy D, Venkateshbabu N, Jamini N, Arathi G ( 2011). Disinfection of dentinal tubules with two different formulations of calcium hydroxide as compared to 2% chlorhexidine: as intracanal medicaments against Enterococcus faecalis and Candida albicans: an in vitro study. J Conserv Dent.

[30] Lakshmi T, Roy A, Merlin ARS ( 2020). Antibacterial activity of achyranthes aspera extract against oral pathogens-an in vitro study. Plant Cell Biotechnol Mol Biol.

[31] Ambulkar S, Tale V, Khilari S ( 2021). Evaluation of the antibacterial potential of traditional medicinal plants against bacteria isolated from dental caries. J Pure Appl Microbiol.

[32] Khan R, Zakir M, Khanam Z, Shakil S, Khan AU ( 2010). Novel compound from Trachyspermum ammi (Ajowan caraway) seeds with antibiofilm and antiadherence activities against Streptococcus mutans: A potential chemotherapeutic agent against dental caries. J Appl Microbiol.

[33] Dadpe MV, Dhore SV, Dahake PT, Kale YJ, Kendre SB, Siddiqui AG ( 2018). Evaluation of antimicrobial efficacy of Trachyspermum ammi (Ajwain) oil and chlorhexidine against oral bacteria: An in vitro study. J Indian Soc Pedod Prev Dent.

[34] Gokhale AB, Damre AS, Kulkarni KR, Saraf MN ( 2002). Preliminary evaluation of anti-inflammatory and anti-arthritic activity of S. lappa, A. speciosa and A aspera. Phytomedicine.

[35] Beegam KS, Joseph A, Singh VP ( 2021). Evaluation of the Antimicrobial Efficacy of Elettaria cardamomum Oil, Trachyspermum ammi Oil and 5% Sodium Hypochlorite Against Enterococcus faecalis Biofilm Formed on Tooth Substrate. Contemp Clin Dent.

[36] Kumar H ( 2013). An in vitro evaluation of the antimicrobial efficacy of Curcuma longa, Tachyspermum ammi, chlorhexidine gluconate, and calcium hydroxide on Enterococcus faecalis. J Conserv Dent.

[37] Kumar RS, Ankola AV, Nagamoti MB, Sankeshwari RM, Sutar KP, Jigan SI, et al ( 2024). Antibacterial and cytotoxicity properties of a polyherbal mouthwash containing Achyranthes aspera and Trachyspermum ammi against selected periodontal pathogens. Journal of Ayurveda and Integrative Medicine.

[38] Kumar RS, Ankola AV, Sankeshwari RM, Hampiholi V, Jalihal S, Khot AJ, et al ( 2024). Clinical and microbial evaluation of mouthwash containing Achyranthes aspera and Trachyspermum ammi: A randomized controlled non-inferiority trial. Journal of Oral Biology and Craniofacial Research.

[39] Kaur GJ, Arora DS ( 2009). Antibacterial and phytochemical screening of Anethum graveolens, Foeniculum vulgare and Trachyspermum ammi. BMC Complement Altern Med.

[40] Mishra P, Sha A, Bhakat P, Mondal S, Mohapatra AK ( 2020). Antibacterial activity assessment of petroleum ether and methanolic extracts of Achyranthes aspera Linn (Amaranthaceae). J Appl Nat Sci.

[41] Pandey G ( 2014). Antioxidant and Antibacterial Activities of Leaf Extract of Achyranthes aspera Linn. (Prickly Chaff Flower). European Journal of Medicinal Plants.

[42] Modareskia M, Fattahi M, Mirjalili MH ( 2022). Thymol screening, phenolic contents, antioxidant and antibacterial activities of Iranian populations of Trachyspermum ammi (L.) Sprague (Apiaceae). Sci Rep.

